# PTH (1-34) enhances the therapeutic effect of bone marrow mesenchymal stem cell-derived exosomes by inhibiting proinflammatory cytokines expression on OA chondrocyte repair in vitro

**DOI:** 10.1186/s13075-022-02778-x

**Published:** 2022-04-29

**Authors:** Li-tao Shao, Liang Luo, Jie-hong Qiu, David Y. B. Deng

**Affiliations:** 1 Department of Scientific Research Center, The Seventh Affiliated Hospital, Sun Yat-Sen University, Shenzhen, China; 2 Department of Critical Care Medicine, The Seventh Affiliated Hospital, Sun Yat-Sen University, Shenzhen, China; 3 The Department of Obstetrics and Gynecology, The Seventh Affiliated Hospital, Sun Yat-sen University, Shenzhen, China

**Keywords:** Osteoarthritis, Parathyroid hormone, Exosome, Chondrocyte

## Abstract

**Background:**

The effects of bone marrow mesenchymal stem cells (BMSCs) during the treatment of cartilage damage have been proven to be attributed to paracrine mechanisms, particularly the effect of exosomes. Exosomes from different batches are inhomogeneous, and different treatment effects are observed between samples. The purpose of this research was to find more effective and homogeneous exosomes for the repair of chondrocytes in osteoarthritis (OA). We observed the potential effects and possible mechanisms of exosomes derived from parathyroid hormone (PTH) (1-34)-preconditioned BMSCs (Exo^PTH^) in the alleviation of OA.

**Materials and methods:**

Exosomes derived from BMSCs (Exo^BMSC^) and Exo^PTH^ were isolated by differential centrifugation. Primary rat chondrocytes were used to establish the OA model by interleukin 1 beta (IL-1β) in vitro. The effects of these two types of exosomes on OA chondrocyte proliferation, migration, apoptosis, and extracellular matrix formation were measured and compared. We observed changes in IL-2, TNF-α, and IL-6 levels via Western blotting (WB), and quantitative real-time PCR (qRT–PCR).

**Results:**

We successfully extracted Exo^BMSC^ and Exo^PTH^ and established an IL-1β-induced OA model in primary chondrocytes from rats. Our study showed that IL-2, TNF-α, and IL-6 levels increased significantly in OA chondrocytes; however, both Exo^BMSC^ and Exo^PTH^ reduced the levels of IL-2, TNF-α, and IL-6. In addition, Exo^PTH^ exhibited stronger anti-inflammatory effects. Exo^PTH^ had a more marked effect on proliferation, migration, and production of the extracellular matrix (Col-II) in OA chondrocytes than Exo^BMSC^ at 24 h.

**Conclusion:**

Exo^PTH^ increased the migration, proliferation, and chondral matrix formation of OA chondrocytes in vitro. In OA chondrocyte therapy, the potential mechanism of Exo^PTH^ might involve the inhibition of production of proinflammatory cytokines. Although the two types of exosomes had some similar effects, most effects of Exo^PTH^ were better than those of Exo^BMSC^, so Exo^PTH^ may have a better ability to alleviate OA.

**Supplementary Information:**

The online version contains supplementary material available at 10.1186/s13075-022-02778-x.

## Introduction

Osteoarthritis (OA) is the most prevalent disease and the leading cause of physical disability [1]. OA affects approximately 265 million people worldwide, and the prevalence of OA is rising due to population aging and the increasing prevalence of obesity [2]. Articular cartilage consists of chondrocytes and extracellular matrix components, which have limited self-regenerative capacity due to the lack of a vasculature and neurons [3]. Currently, there are no effective treatments for patients with OA.

In recent years, bone marrow mesenchymal stem cells (BMSCs) have been a widely applied and effective method for regenerating damaged cartilage [4,5]. However, many challenges are persistent, such as the sources and variation in the quality of BMSCs, complex transplant surgery, and tumor formation [6]. In addition, cell state influence such effects of treatment [7]. Hence, exploration of noncellular agents is urgently needed to protect chondrocytes and delay the progression of OA.

Exosomes have attracted great interest because of their potential value in OA therapy. BMSCs play an important role in the inflammatory response through paracrine mechanisms [4], particularly the effect of exosomes. Exosomes derived from BMSCs (Exo^BMSC^) contain large amounts of active substances, such as DNA, RNA, and proteins [8,9]. Compared with BMSCs therapy, treatment with Exo^BMSC^ exhibits more advantages, including targeted delivery, excellent tissue penetration, outstanding long-term cycling performance, better homing capacity, stable chemical properties, and a substantial capacity for tissue repair and regeneration [10,11,12,13]. Recent studies have demonstrated that OA progression can be ameliorated by Exo^BMSC^ promoting cartilage repair in vivo and vitro [5,7,10]. Exo^BMSC^ protect cartilage by promoting chondrocyte proliferation and maintaining expression of the cartilage matrix [14].

However, the application of Exo^BMSC^ does result in some unwanted reactions [15]. During cartilage regeneration, Exo^BMSC^ can lead to opposing effects, including hypertrophic differentiation and subsequent calcification [7]. Therefore, the identification of more effective and homogeneous exosomes to repair OA chondrocyte lesions is very important. It was reported that parathyroid hormone (PTH) (1-34) enhances the therapeutic effect of BMSCs in cartilage repair [16]. It was previously reported that BMSCs influenced chondrocytes primarily via paracrine mechanisms, particularly exosomes. Therefore, it was also speculated that PTH (1-34) could stimulate BMSCs to produce potent exosomes to regulate chondrocyte metabolism and proliferation.

Thus, we investigated whether exosomes derived from PTH-preconditioned BMSCs (Exo^PTH^) influenced OA-associated chondrocyte damage in vitro.

## Materials and methods

### Characterization of BMSCs and chondrocytes

BMSCs were purchased from Shanghai GuanDao Biological Engineering Co., Ltd. Using flow cytometry, we analyzed BMSC-specific surface markers (CD44, CD90, and CD45) [17]. Briefly, the cell concentration (one million cells/mL) was determined by a cell counting instrument (CountStar®). Then, the cells were incubated with 1% bovine serum albumin (BSA; Gibco) for 30 min. Next, antibodies (CD44, CD90, and CD45) were added to the samples. After incubation for 30 min, the samples were centrifuged at 1000×*g*. The precipitates were washed in PBS and repeated three times. Finally, the samples were identified by flow cytometry. In addition, We also performed the STR testing. Chondrocytes were purchased from Wuhan Procell Life Sci&Tech Co., Ltd. Chondrocytes were confirmed by immunofluorescence (IF) staining for type II collagen (Col-II).

### Extraction and identification of exosomes

First, BMSCs were pretreated with PTH(1-34) for 6 h, then the medium was replaced again, and the medium was collected after 48 h. To harvest exosomes, 200-ml cell culture media were collected. Cells debris was removed from the cell culture supernatant by centrifugation at 300*g* and 2000*g*, and each rotational speed was for 10 min. To remove non-exosome vesicles and any possible apoptotic bodies, the supernatants were then spun at 10,000*g* for 40min. Finally, exosomes were obtained at 120,000 *g* for 70 min. All centrifugations were done at 4 °C. Exosomes were used directly or stored at − 80 °C for follow-up experiments.

Exosomes were analyzed by an HT7700 transmission electron microscope(TEM) (HITACHI, Japan). Protein markers of exosomes were assessed by WB analysis. Antibodies against TSG101 and CD9 (abCAM, USA) were used. The size distribution was detected by nanoparticle tracking analysis (NTA) after dilution 100 times. Primary chondrocytes were treated with exosomes (10 μg/mL) for 24 h, as previously described [18,19].

### IF staining assays

IF staining was performed, as it was previously reported with slight modifications [20]. In brief, chondrocytes were fixed in 4% paraformaldehyde and washed with PBS. Subsequently, the chondrocytes were treated with 0.5% Triton X-100, blocked with 1% bovine serum albumin (BSA) for 30 min, and incubated with primary antibodies overnight at 4 °C. Then, the cells were washed with PBS and incubated with secondary antibodies for 1 h at room temperature. Finally, nuclear counterstaining was performed using DAPI.

### Western blot (WB) assays

WB assays were performed as previously reported with slight modifications [21]. Briefly, samples were lysed in cell lysis buffer, incubated on ice for 5 min, and scraped from the plates. Cell debris was removed by centrifugation. The protein concentrations were measured by a microplate reader. Protein was electrophoresed on a 10% gradient gel at 180 V for approximately 30 min. Then, the proteins were electrically transferred to PVDF membranes. After doing so, the membranes were blocked for 1 h with 5% skim milk at room temperature and subsequently incubated with specific primary antibodies overnight at 4 °C. Following 5×7 min washes in PBS, the membranes were blotted with secondary antibodies for approximately 1 h. The labeled proteins were analyzed with the LAS-3000 Luminescent Image Analyzer (FujiFilm, Tokyo, Japan).

### Cell proliferation assay

EdU staining was measured with an EdU flow cytometry kit. Briefly, the cells were incubated, fixed, and permeabilized following the manufacturer’s protocol. After washing, the EdU-positive cells were detected by flow cytometry.

Cell proliferation was measured via a CCK-8 assay according to the manufacturer’s instructions. Briefly, chondrocytes were seeded onto 96-well plates (2×10^3^ cells/well) and cultured at 37 °C. Chondrocytes were incubated for 4 h before CCK-8 reagents were instilled. Next, 10 μl of CCK-8 solution was added to the 96-well plates. The absorbance was measured with a microplate reader at 450 nm after incubation at 37°C for 1 h.

### Cell migration assay

Cell migration was performed using the scratch assay. When the Cells filled 90–100% confluence in the wells, cells were treated with serum-free DMEM medium for 24 h and a linear scratch was made by a 100-μl sterile pipette tip. Finally, images were acquired using a microscope.

### Apoptosis assay

For the apoptosis assays, 3×10^5^cells were collected from each sample, rinsed with PBS, and resuspended in 200μl 1× Binding Buffer. After doing so, Annexin 5μl Annexin V-FITC and 5 μl Propidium Iodide (PI) solution was added for 10 min at room temperature. Finally, the cells were analyzed on a flow cytometer.

### Cytokine antibody array

Cytokine expression in chondrocytes was detected using Quantibody® Rat Inflammation Array (RayBiotech, China) and quantified following the manufacturer’s instructions. Briefly, the cells were incubated with a standard protein cocktail, biotinylated antibody cocktail, and Cy3 equivalent dye labeled-streptavidin. Finally, scan and perform data extraction and analysis.

### Total RNA extraction and quantitative real-time PCR (qRT-PCR)

In short, total RNA was extracted using RNA-Quick Purification Kit (Shanghai Yishan Biotechnology Co, Ltd, China). First-strand cDNA was synthesized using HiScript II One Step RT-PCR Kit (Tiangen, China). The primers used to amplify the indicated genes are listed in Supplementary Table 1. Finally, using ChamQ SYBR qPCR Master Mix (Tiangen, China) performed qRT-PCR.

## Results

### Characterization of BMSCs and chondrocytes

The BMSCs were observed by an inverted microscope (Figure Supplementary 1A). Most BMSCs were negative for CD45 and positive for CD44 and CD90 (Figures Supplementary 1B-D). These features were observed by flow cytometry analysis. STR genotyping was performed by Shanghai GuanDao Biological Engineering Co., Ltd. (Figure Supplementary 1E). Chondrocytes were purchased from Wuhan Procell Life SCI&TECH Co., Ltd. Chondrocytes were identified by Col-II (IF) (Figure Supplementary 1F).

### Identification of Exo^BMSC^ and Exo^PTH^

**Fig. 1:**
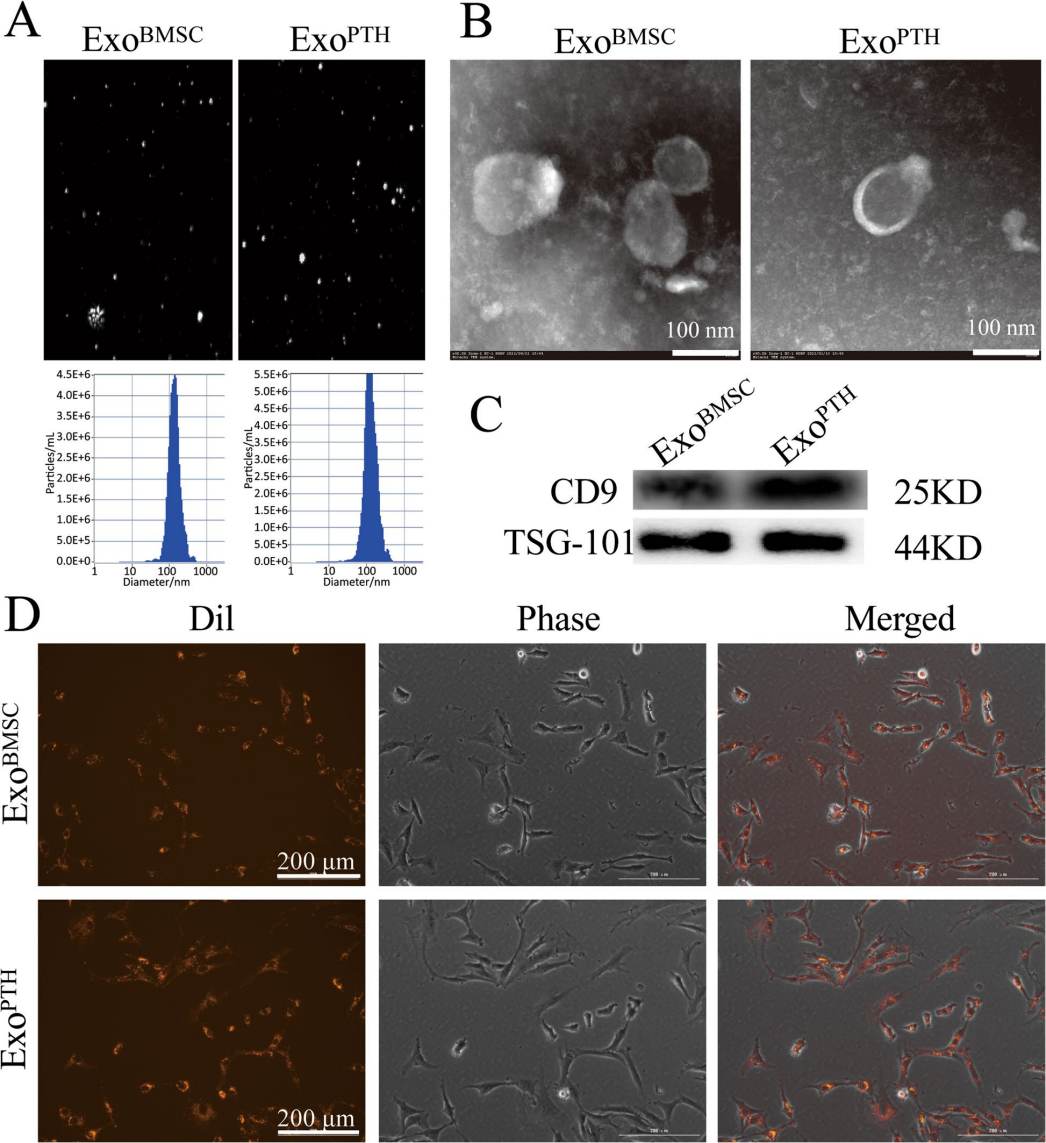
Characterization of Exo^BMSC^ and Exo^PTH^. **A**. Size distribution of Exo^BMSC^ and Exo^PTH^ is indicated by nanoparticle tracking analysis. **B** Representative transmission electron microscopy images of Exo^BMSC^ and Exo^PTH^ (scale bar = 100 nm). **C** Positive staining for the exosome markers CD9 and TSG101 was observed for Exo^BMSCs^ and Exo^PTH^, as determined by Western blotting. **D** Dil-labeled exosomes (red fluorescence) were used to observe exosome uptake by chondrocytes. Exo^BMSC^: Exosomes derived from BMSCs; Exo^PTH^: exosomes derived from PTH-preconditioned BMSCs; BMSCs: bone marrow mesenchymal stem cells

The Exo^BMSC^ and Exo^PTH^ particles were mostly in the 60 to 150 nm range (Fig. 1A), as indicated by NTA. For SEM observation, the results showed that Exo^BMSC^ and Exo^PTH^ exhibited a saucer or cup shape, which is a typical morphology for exosomes (Fig. 1B). The results of CD9 and TSG101 are specifically expressed by WB analysis in the exosomes (Fig. 1C). Exosomes labeled with Dil are shown in dotted red. We observed that the distribution of exosomes was in the cytosolic domain of the chondrocytes (Fig. 1D), indicating the successful uptake of Exo^BMSC^ and Exo^PTH^ by chondrocytes.

### Selection of the concentration of IL-1β / PTH (1-34)

The migration ratio of chondrocytes was significantly inhibited by IL-1β at concentrations of 20 and 50 ng/ml. Compared with the control, 1 ng/ml group, and 5 ng/ml group, 20 ng/ml group significantly decreased (78.97%, 77.76%, and 72.71% respectively), and 50 ng/ml significantly decreased (84.21%, 97.02%, and 79.51% respectively). Compared with the control and 1 ng/ml groups, the migration ratio of the 10 ng/ml group was significantly decreased (76.35% and 74.99%). However, there was no difference between the 10, 20, and 50 ng/ml groups. Therefore, we chose 20 ng/ml as the concentration of IL-1β (Figure Supplementary 2A, D). Chondrocytes and BMSCs were cultured with various concentrations of PTH (1-34) and compared with the controls. The migration ratio of the PTH (1-34) 10 nM/ml group in chondrocytes and BMSCs was significantly higher than that in the other groups. Compared with the control, 1 nM/ml, 5 nM/ml, 20 nM/ml and 50 nM/ml groups, the migration ratio of the PTH (1-34) 10 nM/ml group in BMSCs was significantly increased (28.02%, 26.55%, 28.95%, 21.23%, and 54.88% respectively) (Figures Supplementary 2B, E). Compared with the control, 1 nM/ml, 5 nM/ml, 20 nM/ml and 50 nM/ml groups, the migration ratio of the PTH (1-34) 10 nM/ml group in chondrocytes was significantly increased (46.95%, 48.14%, 42.42%, 79.66%, and 111.72% respectively) (Figures Supplementary 2C, F).

### The effects of Exo^BMSC^ and Exo^PTH^ on chondrocytes

We observed the cell viability of chondrocytes treated with Exo^BMSC^ and Exo^PTH^ via the scratch and CCK-8 assay in vitro. As shown in Figures Supplementary 3A-B, the scratch assay data showed that the migration ratio of chondrocytes was promoted by Exo^BMSC^ and Exo^PTH^. Compared with the Exo^BMSC^ and Exo^PTH^ groups, the migration ratio of the control group significantly decreased (46.45% and 61.07%). Moreover, the Exo^PTH^ treatment showed stronger promotion than the Exo^BMSC^ treatment. Compared with the Exo^BMSC^ group, the Exo^PTH^ group significantly increased by 37.56%. The proliferation of chondrocytes is shown in Figure Supplementary 3C, after 48 h, both Exo^BMSC^ and Exo^PTH^ promoted chondrocyte proliferation, and Exo^PTH^ showed stronger promotion than Exo^BMSC^.

### The effects of Exo^BMSC^ and Exo^PTH^ on OA chondrocyte migration and proliferation

**Fig. 2:**
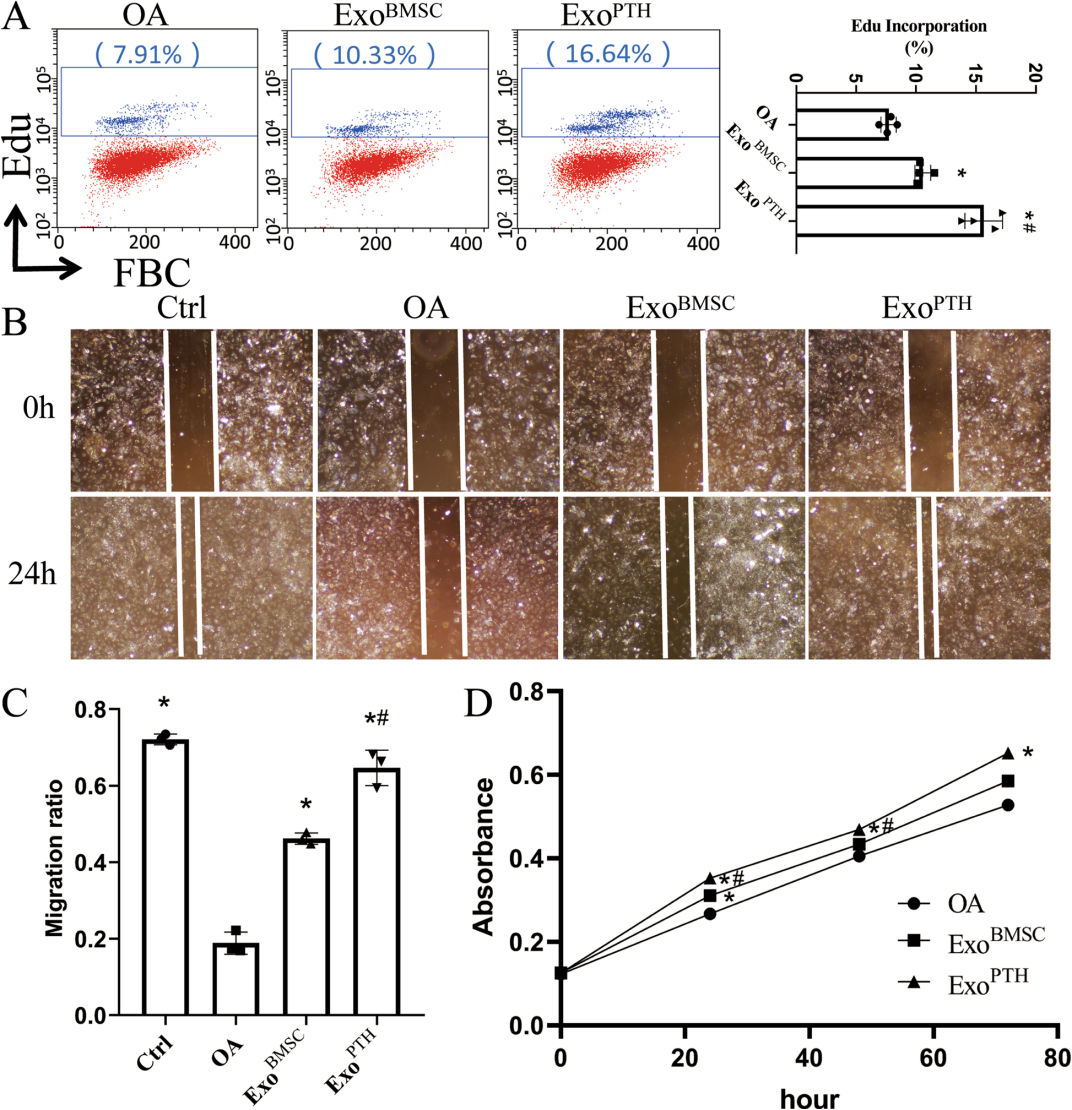
The effect of Exo^BMSC^ and Exo^PTH^ on OA chondrocyte proliferation was assayed by a flow cytometry, a scratch migration assay, and CCK-8 assay. **A** Flow cytometry was used to explore the cell proliferation of the OA, Exo^BMSC^, and Exo^PTH^ groups by the EdU flow cytometry kit. **B** A scratch wound-healing migration assay was performed with control, OA, Exo^BMSC^, and Exo^PTH^ treatments. **C** Statistical results of the scratch wound-healing migration assay. **D** The cell proliferation ability of the control, OA, Exo^BMSC^, and Exo^PTH^ groups was detected by a CCK-8 assay. Data are presented as the mean ± SD, *p < 0.05 versus OA group and ^#^*p* < 0.05 versus Exo^BMSC^ group. Ctrl, control; OA, osteoarthritis; Exo^BMSC^, exosomes derived from BMSCs; Exo^PTH^, exosomes derived from PTH-preconditioned BMSCs; BMSCs, bone marrow mesenchymal stem cells

OA chondrocyte proliferation was assessed by flow cytometry (Fig. 2A). Compared with the Exo^BMSC^ and Exo^PTH^ groups, the rate of OA chondrocyte proliferation decreased significantly by 27.06% and 50.73%. Compared with the Exo^BMSC^ group, the Exo^PTH^ group significantly increased by 48.06%.

As shown in Fig. 2B, C, the migration ratio of chondrocytes was significantly inhibited in the OA group. Compared with the Exo^BMSC^ and Exo^PTH^ groups, the migration ratio of OA chondrocytes decreased significantly (59.52% and 70.82%). Moreover, the Exo^PTH^ treatment was more effective than the Exo^BMSC^ treatment. Compared with the Exo^BMSC^ group, the Exo^PTH^ group significantly increased by 39.96%.

We observed the viability of OA chondrocytes treated with Exo^BMSC^ and Exo^PTH^ via a CCK-8 assay (Fig. 2D). Both Exo^BMSC^ and Exo^PTH^ promoted OA chondrocyte proliferation after 24 h, 48 h, and 72 h. Moreover, Exo^PTH^ was more effective than Exo^BMSC^.

**Fig. 3:**
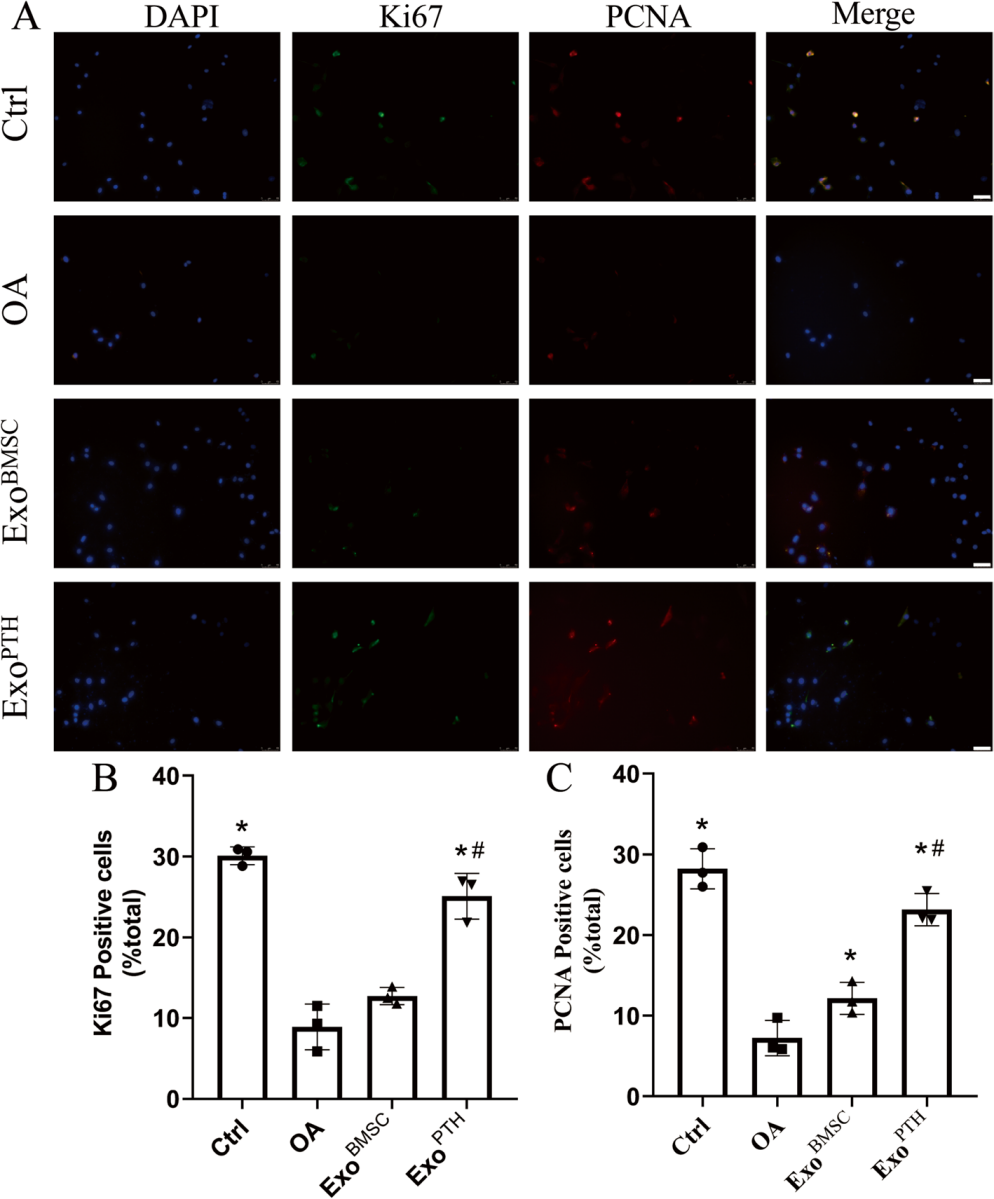
The effect of Exo^BMSC^ and Exo^PTH^ on OA chondrocyte proliferation was assayed by immunofluorescence staining for ki67 and PCNA. **A** Immunofluorescence staining for ki67 and PCNA. **B** The positive expression ratio of ki67. **C** The positive expression ratio of PCNA. Data are presented as the mean ± SD, *p < 0.05 versus OA group and ^#^*p* < 0.05 versus Exo^BMSC^ group. Bars = 50 μm. Ctrl, control; OA, osteoarthritis; Exo^BMSC^, exosomes derived from BMSCs; Exo^PTH^, exosomes derived from PTH-preconditioned BMSCs; BMSCs, bone marrow mesenchymal stem cells

The Ki67 index (percentage of Ki67-positive cells/total cells) was calculated (Fig. 3). Compared with the control, Exo^BMSC^, and Exo^PTH^ groups, the percentage of Ki67-positive cells was significantly decreased in the OA group (70.44%, 30.03%, and 64.51%, respectively). The percentage of Ki67-positive cells is significantly increased by 97.17% in the Exo^PTH^ group than in the Exo^BMSC^ group.

The PCNA index (percentage of PCNA-positive cells/total cells) was calculated (Fig. 3). Compared with the control, Exo^BMSC^, and Exo^PTH^ groups, the percentage of PCNA-positive cells was significantly decreased in the OA group. Compared with the control, Exo^BMSC^, and Exo^PTH^ groups, the percentage of PCNA-positive cells was significantly decreased in the OA group (74.34%, 40.46%, and 68.74%, respectively). The percentage of PCNA-positive cell was significantly lower in the Exo^BMSC^ group than the Exo^PTH^ group (Exo^PTH^ group significantly increased by 47.50%)

### The apoptotic effects of Exo^BMSC^ and Exo^PTH^ on OA chondrocytes

**Fig. 4:**
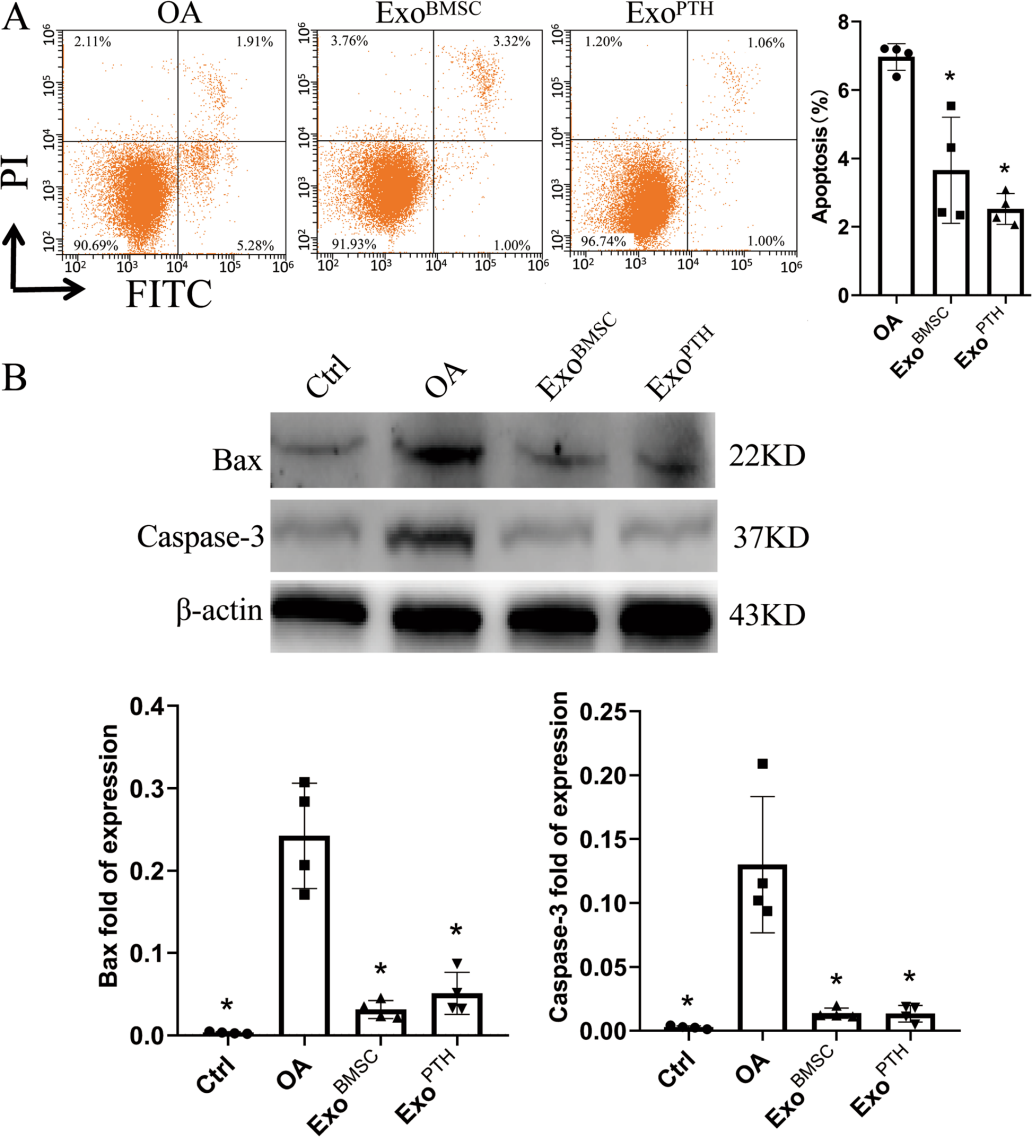
The effect of Exo^BMSC^ and Exo^PTH^ on OA chondrocyte apoptosis was determined by flow cytometry and Western blotting. **A** Cells were processed by flow cytometry using Annexin V-FITC/PI staining. The percentage of the Annexin V-positive population indicates apoptosis induction in each group. **B** Cell lysates were prepared and analyzed by Western blotting for Bax and Caspase-3. β-actin was used as a loading control. Band intensities were quantified by IPP and normalized to β-actin. Data are presented as the mean ± SD, *p < 0.05 versus the OA group. Ctrl, control; OA, osteoarthritis; Exo^BMSC^, exosomes derived from BMSCs; Exo^PTH^, exosomes derived from PTH-preconditioned BMSCs; BMSCs, bone marrow mesenchymal stem cells

OA chondrocyte apoptosis was assessed by flow cytometry (Fig. 4A). The rate of OA chondrocyte apoptosis increased significantly in the Exo^BMSC^ and Exo^PTH^ groups, but no significant differences were detected in the Exo^BMSC^ and Exo^PTH^ groups. Compared with the Exo^BMSC^ and Exo^PTH^ groups, the rate of OA chondrocyte apoptosis increased significantly by 90.44% and 176.59%. However, there were no significant differences between the Exo^PTH^ group and the Exo^BMSC^ group.

Conformational changes were analyzed by WB using Bax and Caspase-3 antibodies (Fig. 4B). In the OA group, Bax and Caspase-3 expression levels were significantly higher than those in the Exo^PTH^ group and the Exo^BMSC^ group (the Exo^PTH^ group significantly decreased [78.98% and 89.78%]; the Exo^BMSC^ group significantly decreased [87.08% and 89.39%]), but no significant differences were detected in the Bax and Caspase-3 in the Exo^BMSC^ and Exo^PTH^ groups.

### The effects of Exo^BMSC^ and Exo^PTH^ on the extracellular matrix in OA chondrocytes

Col-II and MMP-13 expression were observed by WB and IF, as shown in Figure Supplementary 4. By WB analysis, compared with the OA group, Col-II expression of control (significantly increased [92.54%]) and Exo^PTH^ groups (significantly increased [91.75%]) were significantly upregulated. Col-II expression was significantly downregulated in the OA group compared with the other three groups by IF analyses (OA vs control group, OA group significantly decreased [91.79%]; OA vs Exo^BMSC^ group, OA group significantly decreased [86.10%]; OA vs Exo^PTH^ group, OA group significantly decreased [96.14%]). The Exo^PTH^ group displayed significantly higher Col-II expression than the Exo^BMSC^ group (Exo^PTH^ vs Exo^BMSC^ group, Exo^PTH^ group significantly increased [260.29%]). By WB analysis, compared with the OA group, MMP-13 expression of control (significantly decreased [77.42%] and Exo^PTH^ groups (significantly decreased [85.56%]) were significantly downregulated. By IF analysis, compared with the OA group, MMP-13 expression of control (significantly decreased [89.22%] and Exo^PTH^ groups (significantly decreased [89.48%]) were significantly downregulated. However, no significant difference was observed between the OA and Exo^BMSC^ groups.

### The effects of Exo^BMSC^ and Exo^PTH^ on inflammatory cytokines in OA chondrocytes

**Fig. 5:**
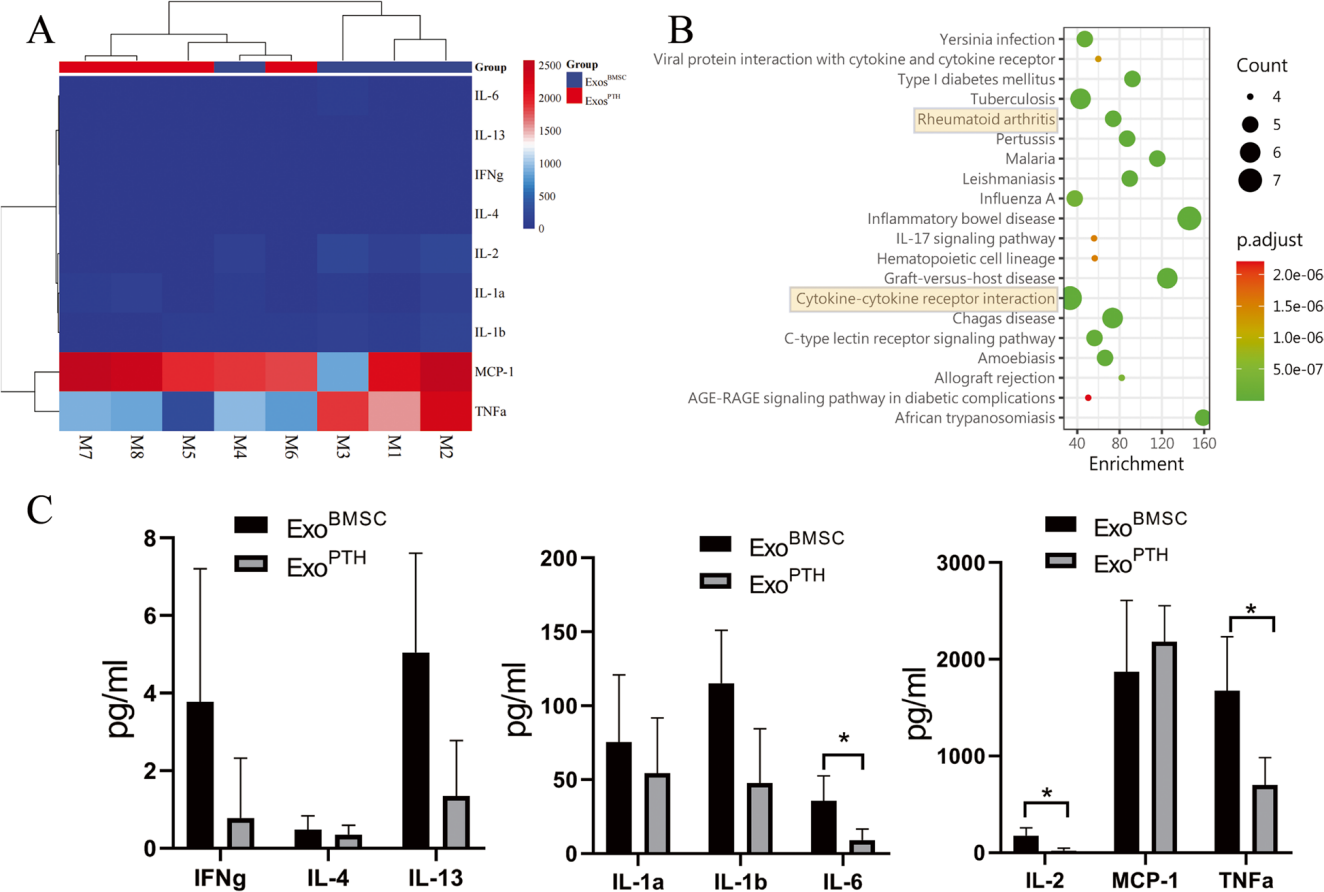
The effects of Exo^BMSC^ and Exo^PTH^ on inflammatory cytokines in OA chondrocytes were detected by an inflammation array. **A** Gene-level exploratory analysis and heatmaps of differentially expressed genes in the Exo^BMSC^ and Exo^PTH^ groups; (M1–M4): Exo^BMSC^ group; (M5–M8): Exo^PTH^ group. **B** KEGG functional enrichment in KEGG pathways was analyzed. **C** Statistical results of the inflammation array. Data are presented as the mean ± SD, *p < 0.05 (Exo^PTH^ versus Exo^BMSC^ group). Exo^BMSC^, Exosomes derived from BMSCs; Exo^PTH^, exosomes derived from PTH-preconditioned BMSCs; BMSCs, bone marrow mesenchymal stem cells

Using Rat Inflammation Cytokine Antibody Array, we detected the expression level of inflammatory factors in both the Exo^BMSC^ group and the Exo^PTH^ group (Fig. 5). IL-2, TNF-α, and IL-6 expression levels were significantly lower in the Exo^PTH^ group than in the Exo^BMSC^ group (Exo^PTH^ group significantly decreased [88.04%, 74.71%, and 58.06%, respectively]). However, IL-1a, IL-1b, IL-4, IL-13, MCP-1, and IFNg were not different between the two groups.

**Fig. 6:**
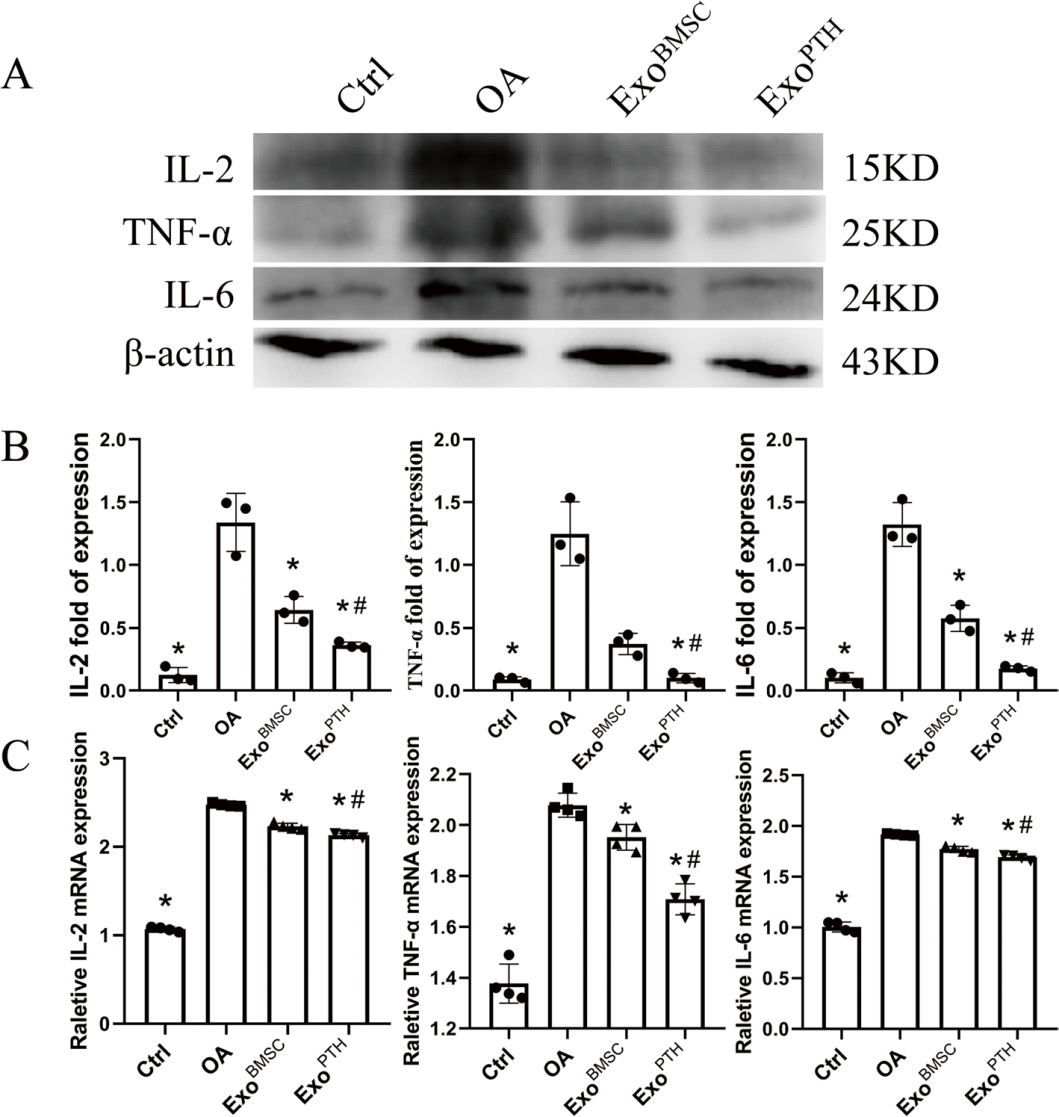
The results obtained for inflammatory factors (IL-2/TNF-α/IL-6) were verified by Western blotting and qRT-PCR. **A** Western blotting assay for IL-2/TNF-α/IL-6 in each group. **B** Statistical results of the Western blotting assay (IL-2/TNF-α/IL-6). **C** qRT-PCR assay for IL-2/TNF-α/IL-6 in each group

IL-2, TNF-α, and IL-6 expression were detected by WB (Fig. 6A, B). By WB analysis, compared with the OA group, IL-2 expression of the control (significantly decreased [90.95%]), Exo^BMSC^ (significantly decreased [52.67%]), and Exo^PTH^ groups (significantly decreased [72.93%]) were significantly downregulated. The TNF-α expression levels were significantly lower in the control, Exo^BMSC^, and Exo^PTH^ than in the OA group (control, Exo^BMSC^, and Exo^PTH^ group significantly decreased [92.84%, 70.14%, and 91.99%, respectively]). The IL-6 expression levels were significantly lower in the control, Exo^BMSC^, and Exo^PTH^ than in the OA group (control, Exo^BMSC^, and Exo^PTH^ group significantly decreased [92.22%, 56.42%, and 86.67%, respectively]). The Exo^PTH^ group displayed significantly lower IL-2, TNF-α, and IL-6 expression than the Exo^BMSC^ group (Exo^PTH^ group significantly decreased [43.69%, 73.16%, and 69.43%, respectively]).

IL-2, TNF-α, and IL-6 expression were detected by qRT-PCR (Fig. 6C). By qRT-PCR analysis, compared with the OA group, IL-2 expression of the control (significantly decreased [35.08%]), Exo^BMSC^ (significantly decreased [2.79%]), and Exo^PTH^ groups (significantly decreased [15.02%]) were significantly downregulated. The TNF-α expression levels were significantly lower in the control, Exo^BMSC^, and Exo^PTH^ than in the OA group (control, Exo^BMSC^, and Exo^PTH^ group significantly decreased [33.75%, 6.07%, and 17.80%, respectively]). The IL-6 expression levels were significantly lower in the control, Exo^BMSC^, and Exo^PTH^ than in the OA group (control, Exo^BMSC^, and Exo^PTH^ group significantly decreased [47.53%, 7.58%, and 11.75%, respectively]). The Exo^PTH^ group displayed significantly lower IL-2, TNF-α, and IL-6 expressions than the Exo^BMSC^ group (Exo^PTH^ group significantly decreased [12.58%, 12.49%, and 4.52%, respectively]).

## Discussion

In the present study, we found that both Exo^BMSC^ and Exo^PTH^ enhanced the chondrogenic phenotype of chondrocytes, but Exo^PTH^ appeared to have better performance.

OA, which seriously influences the quality of life of patients, is influenced by many factors. The most important feature of OA is articular cartilage degeneration, which leads to the inhibition of cell proliferation, migration, and matrix production [22]. OA therapies are of widespread interest, but OA therapy is limited to symptomatic treatment at present. Due to the complexity of OA pathogenesis, the transplantation of BMSCs may be the best therapeutic option to date [23,24]. However, many questions remain open, such as the variable sources and quality of BMSCs, the complexity of transplant surgery, and the risk of tumor formation [6]. Currently, there is a widely accepted point of view that Exo^BMSC^ have a positive impact on OA, but Exo^BMSC^ suffer from low efficiency as well as concerns about heterogeneity [7]. PTH induces BMSC differentiation into chondrocytes and enhances repair after articular cartilage injury [16,25,26,27]. Thus, we investigated whether the effect of Exo^BMSC^ could be improved by extrinsic PTH (1-34) in our study.

To induce a model of OA in primary chondrocytes, we treated the cells with topical IL-1β, which has become a widely accepted method [28,29]. According to a cell migration experiment and the available literature [30], 20 ng/ml IL-1β was selected as the optimal concentration. The proliferation and migration of chondrocytes decreased significantly, and the apoptosis of chondrocytes obviously increased after stimulation with IL-1β. This finding was subsequently confirmed by many experiments [31,32]. As we observed in the present study, IL-2, TNF-α, and IL-6 were increased in OA chondrocyte, which is consistent with some previous studies from other groups [33,34]. These results demonstrate that the in vitro model of OA chondrocytes used in the following experiments was established successfully, which is consistent with previous studies.

In recent years, many studies have shown that exosomes can arrest the progression of cartilage destruction in OA. Exosomes regulate various biological processes by transferring microRNAs, proteins, and other nucleic acids to other cells. Lei He et al. provided evidence that Exo^BMSC^ treatment significantly attenuated the inhibitory effect of IL-1β on the proliferation and migration of chondrocytes and IL-1β-induced downregulation of Col-II and upregulation of MMP-13 [10,35]. These effects of Exo^BMSC^ on OA chondrocytes are compatible with our findings. More importantly, we discovered that Exo^PTH^ has a more marked effect on the proliferation, migration, and production of extracellular matrix (Col-II) than Exo^BMSC^ in OA chondrocytes. Unfortunately, in comparison to Exo^BMSC^, Exo^PTH^ does not significantly inhibit apoptosis or MMP-13 expression in OA chondrocytes. This is likely due to two main factors. A limitation is that our testing was performed at only one time point, and another limitation is that we used only one concentration of Exo^PTH^. Therefore, our results reveal that Exo^PTH^ may play an important role in preventing OA progression by promoting proliferation, migration, and production of the extracellular matrix (Col-II) in OA chondrocytes but not by inhibiting apoptosis and MMP-13 expression.

**Fig. 7:**
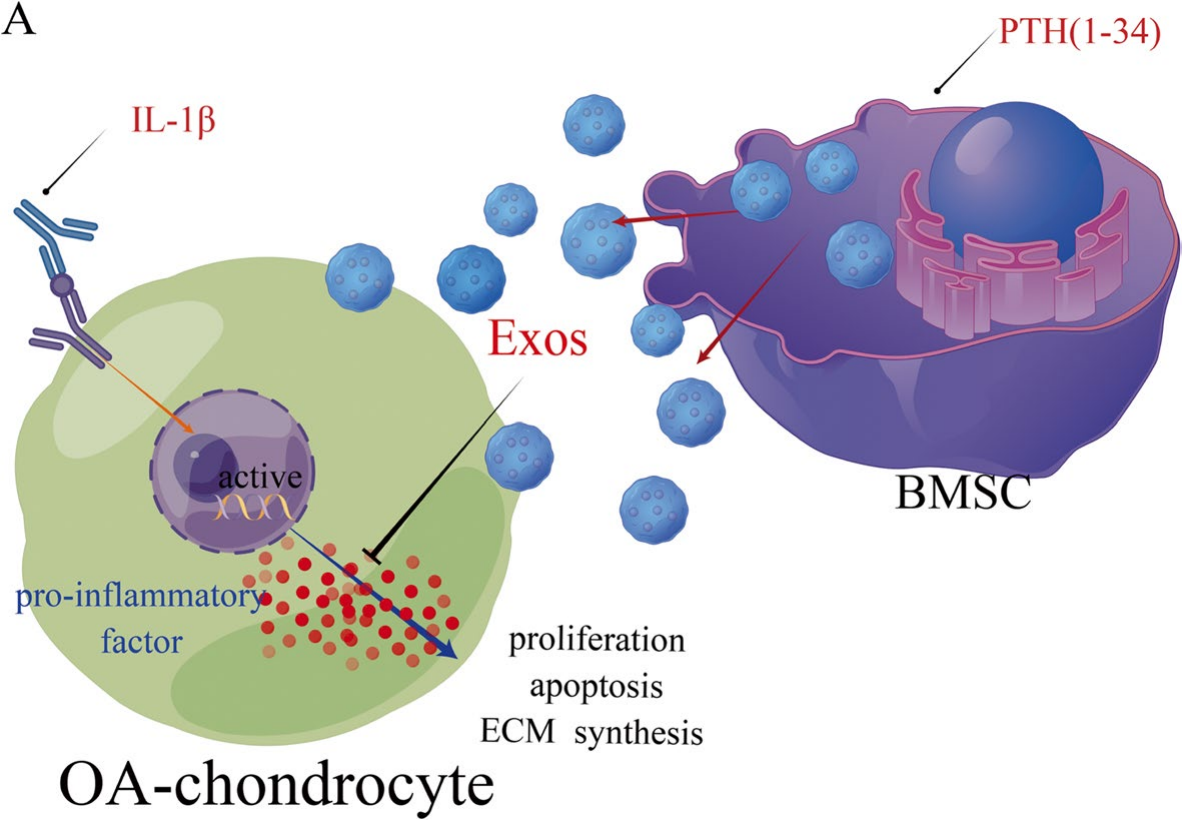
**A** Mechanistic diagram of our study. IL-1β, interleukin-1β; PTH, parathyroid hormone; OA, osteoarthritis; Exos, exosomes derived from PTH-preconditioned BMSCs; BMSCs, bone marrow mesenchymal stem cells; ECM, extracellular matrix

A number of factors can influence OA chondrocytes. Exo^BMSC^ and Exo^PTH^ could alter the state of OA chondrocytes, but the mechanism of this process remains poorly understood. In many studies, the proinflammatory cytokine (IL-2, TNF-α, and IL-6) has been identified as playing an important role in inflammatory effects by controlling the proliferation and apoptosis of chondrocytes [36,37,38,39]. It has been reported that chondrocytes are impaired because of excess IL-2, TNF-α, and IL-6 leading to disruption of homeostasis between anabolism and catabolism of the extracellular matrix [40,41,42,43]. In the present study, compared with that in the OA group, IL-2, TNF-α, and IL-6 expression was decreased in the Exo^BMSC^ and Exo^PTH^ groups. Moreover, Exo^PTH^ has a stronger ability to inhibit IL-2, TNF-α, and IL-6 production. Thus, Exo^PTH^ may exert stronger effects on proliferation, migration, and extracellular matrix production by decreasing IL-2, TNF-α, and IL-6 expression in OA chondrocytes. The possible mechanism of Exo^PTH^ on OA chondrocytes is shown in Fig. 7.

Although our study showed that Exo^PTH^ plays an important role in OA chondrocyte repair and chondral matrix formation, there are still several limitations that are worth noting. Exosome contents, which contain microRNAs, mRNAs, lncRNAs, and even various proteins, play a major role in their regulatory function. A large number of proteomic and RNA cargos in Exo^BMSC^ and Exo^PTH^ are unknown. Second, the interval for joint injections and optimum concentration of Exo^PTH^ need to be explored. Third, the possible mechanisms which may affect cartilage homeostasis are numerous [24], we only studied the changes of some inflammatory factors. Despite several limitations of this study, several nanomedicines based on the results of previous studies are potentially useful in the treatment of arthritis [44,45,46]. Therefore, we went on to explore potential mechanisms of Exo^PTH^ in the future, which may provide a novel idea for the early treatment of OA.

## Conclusion

In the present study, we found that Exo^PTH^ could enhance the proliferation, migration, and chondral matrix formation of OA chondrocytes induced by IL-1β. Moreover, the potential mechanism of Exo^PTH^ in OA therapy might involve the inhibition of the production of proinflammatory factors (IL-2, TNF-α, and IL-6). Although these types of exosomes have some similar effects, Exo^PTH^ had better effects than Exo^BMSC^, so Exo^PTH^ may play a more important role in the alleviation of OA. In conclusion, we conclude that Exo^PTH^ may be effective in OA treatment, and investigation of intra-articular injections of Exo^PTH^ could shed new light on their clinical application.

## Supplementary Information

**Additional file 1: Figure Supplementary 1.** BMSCs and chondrocytes were identified. (A). Morphology of BMSCs observed by inverted microscopy. (B-D). BMSCs were authenticated by flow cytometry. (E). BMSCs were authenticated by STR DNA profiling. (F) Immunofluorescence staining was used to identify chondrocytes. BMSCs: bone marrow mesenchymal stem cells. Ch: chondrocytes.

**Additional file 2: Figure Supplementary 2.** The concentrations of IL-1β and PTH (1-34) were chosen. (A). A series of IL-1β concentrations were investigated in chondrocytes. (B). Different concentrations of PTH (1-34) were investigated in BMSCs. (C). Different concentrations of PTH (1-34) were investigated in chondrocytes. (D). Statistical results of IL-1β concentrations. *p < 0.05 versus the control group, ^#^*p* < 0.05 versus the 1 ng/ml group and ^&^*p* < 0.05 versus the 5 ng/ml group. (E). Statistical results of different concentrations of PTH (1-34) acted on BMSCs. *p < 0.05 versus 10 nM/ml group and ^#^*p* < 0.05 versus 50 nM/ml group. (F). Statistical results showing the effects of different concentrations of PTH (1-34) acted on chondrocytes. *p < 0.05 versus the 10 nM/ml group and ^#^*p* < 0.05 versus 50 nM/ml group. IL-1β: interleukin-1β; PTH: parathyroid hormone; OA: osteoarthritis; BMSCs: bone marrow mesenchymal stem cells.

**Additional file 3: Figure Supplementary 3.** The effect of Exo^BMSC^ and Exo^PTH^ on chondrocyte proliferation was assayed by the scratch wound-healing migration assay and CCK-8. (A). The scratch wound-healing migration assay was performed with control, Exo^BMSC^, and Exo^PTH^ groups. (B). Statistical results of the migration ratio. (C). The cell proliferation ability of the control, Exo^BMSC^ and Exo^PTH^ groups was detected by a CCK-8 assay. Data are presented as the mean ± SD, *p < 0.05 versus the control group and ^#^*p* < 0.05 versus the Exo^BMSC^ group. Exo^BMSC^: exosomes derived from BMSCs; Exo^PTH^: exosomes derived from PTH-preconditioned BMSCs.

**Additional file 4: Figure Supplementary 4:** The effect of Exo^BMSC^ and Exo^PTH^ on the OA chondrocyte extracellular matrix was detected by Western blot and immunofluorescence. (A). Western blot assay for collagen-II (Col-II) and matrix metalloproteinase-13 (MMP-13) in each group. (B). Immunofluorescence assay for Col-II and MMP-13 in each group. Data are presented as the mean ± SD, *p < 0.05 versus the OA group and ^#^*p* < 0.05 versus the Exo^BMSC^ group. Bars = 50 μm; OA: osteoarthritis; Exo^BMSC^: exosomes derived from BMSCs; Exo^PTH^: exosomes derived from PTH-preconditioned BMSCs; BMSCs: bone marrow mesenchymal stem cells.

**Additional file 5: Table 1.** List of the three PCR Primer Pairs chosen for *in vitro* PCR validation.

## Data Availability

All data supporting our findings within the manuscript
