# Supplementary figures and images for: PTH (1-34) enhances the therapeutic effect of bone marrow mesenchymal stem cell-derived exosomes by inhibiting proinflammatory cytokines expression on OA chondrocyte repair in vitro
Source: Arthritis Res Ther. 2022 Apr 29;24:96. doi: 10.1186/s13075-022-02778-x (PMC9052609; doi:10.1186/s13075-022-02778-x)

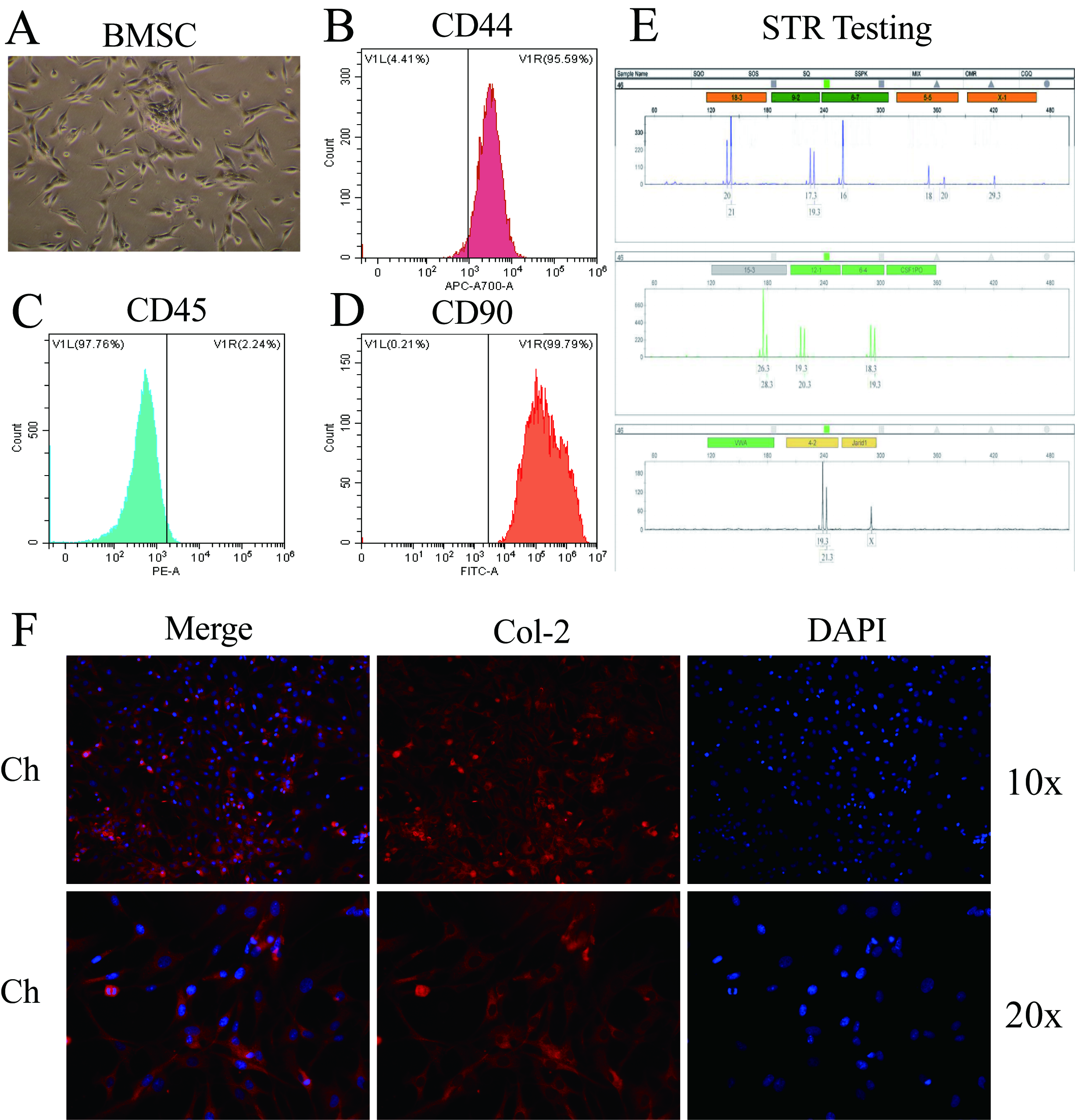

Supplement: Supplementary file 1 — Additional file 1: Figure Supplementary 1. BMSCs and chondrocytes were identified. (A). Morphology of BMSCs observed by inverted microscopy. (B-D). BMSCs were authenticated by flow cytometry. (E). BMSCs were authenticated by STR DNA profiling. (F) Immunofluorescence staining was used to identify chondrocytes. BMSCs: bone marrow mesenchymal stem cells. Ch: chondrocytes. [file 13075_2022_2778_MOESM1_ESM.tif]

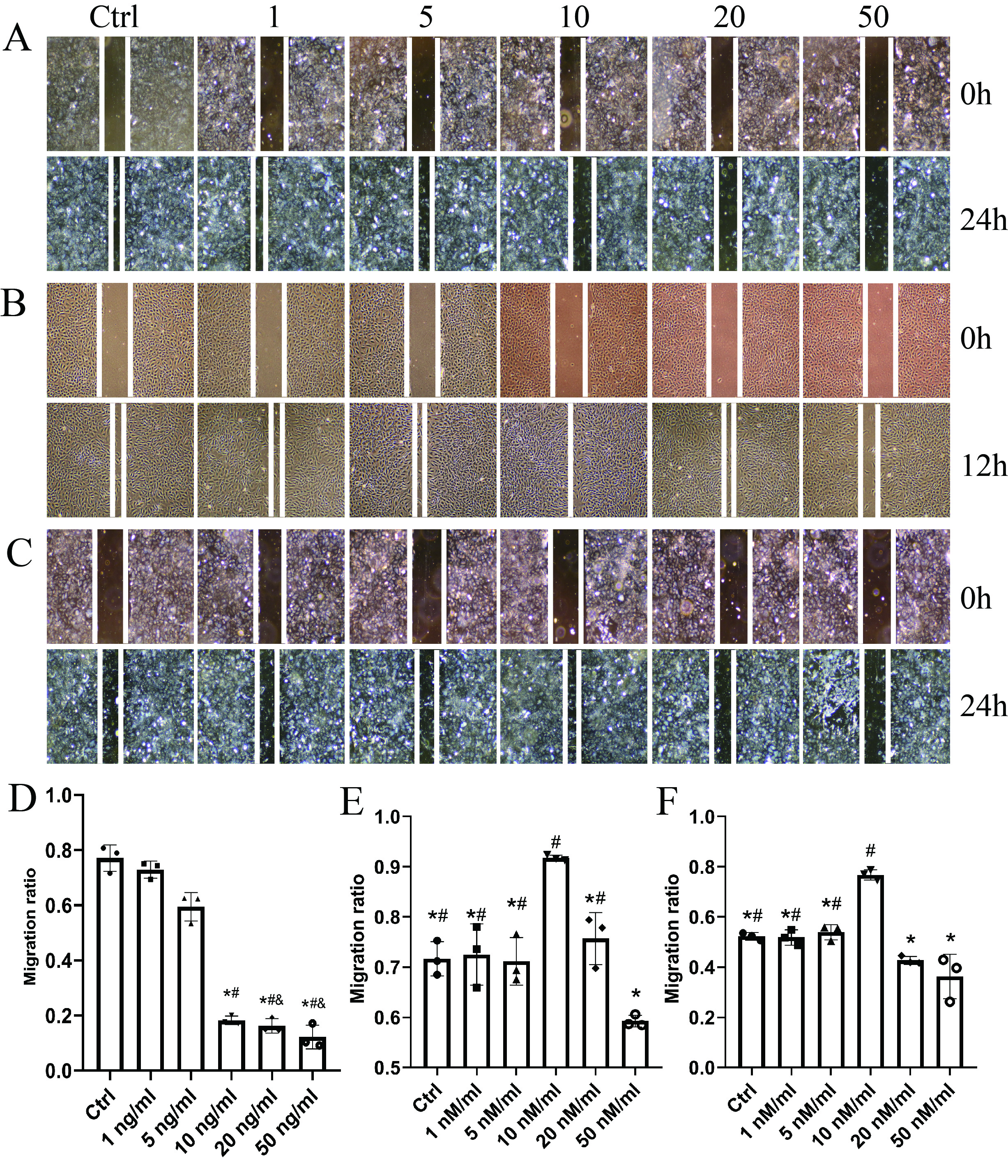

Supplement: Supplementary file 2 — Additional file 2: Figure Supplementary 2. The concentrations of IL-1β and PTH (1-34) were chosen. (A). A series of IL-1β concentrations were investigated in chondrocytes. (B). Different concentrations of PTH (1-34) were investigated in BMSCs. (C). Different concentrations of PTH (1-34) were investigated in chondrocytes. (D). Statistical results of IL-1β concentrations. *p < 0.05 versus the control group, #p < 0.05 versus the 1 ng/ml group and &p < 0.05 versus the 5 ng/ml group. (E). Statistical results of different concentrations of PTH (1-34) acted on BMSCs. *p < 0.05 versus 10 nM/ml group and #p < 0.05 versus 50 nM/ml group. (F). Statistical results showing the effects of different concentrations of PTH (1-34) acted on chondrocytes. *p < 0.05 versus the 10 nM/ml group and #p < 0.05 versus 50 nM/ml group. IL-1β: interleukin-1β; PTH: parathyroid hormone; OA: osteoarthritis; BMSCs: bone marrow mesenchymal stem cells. [file 13075_2022_2778_MOESM2_ESM.jpg]

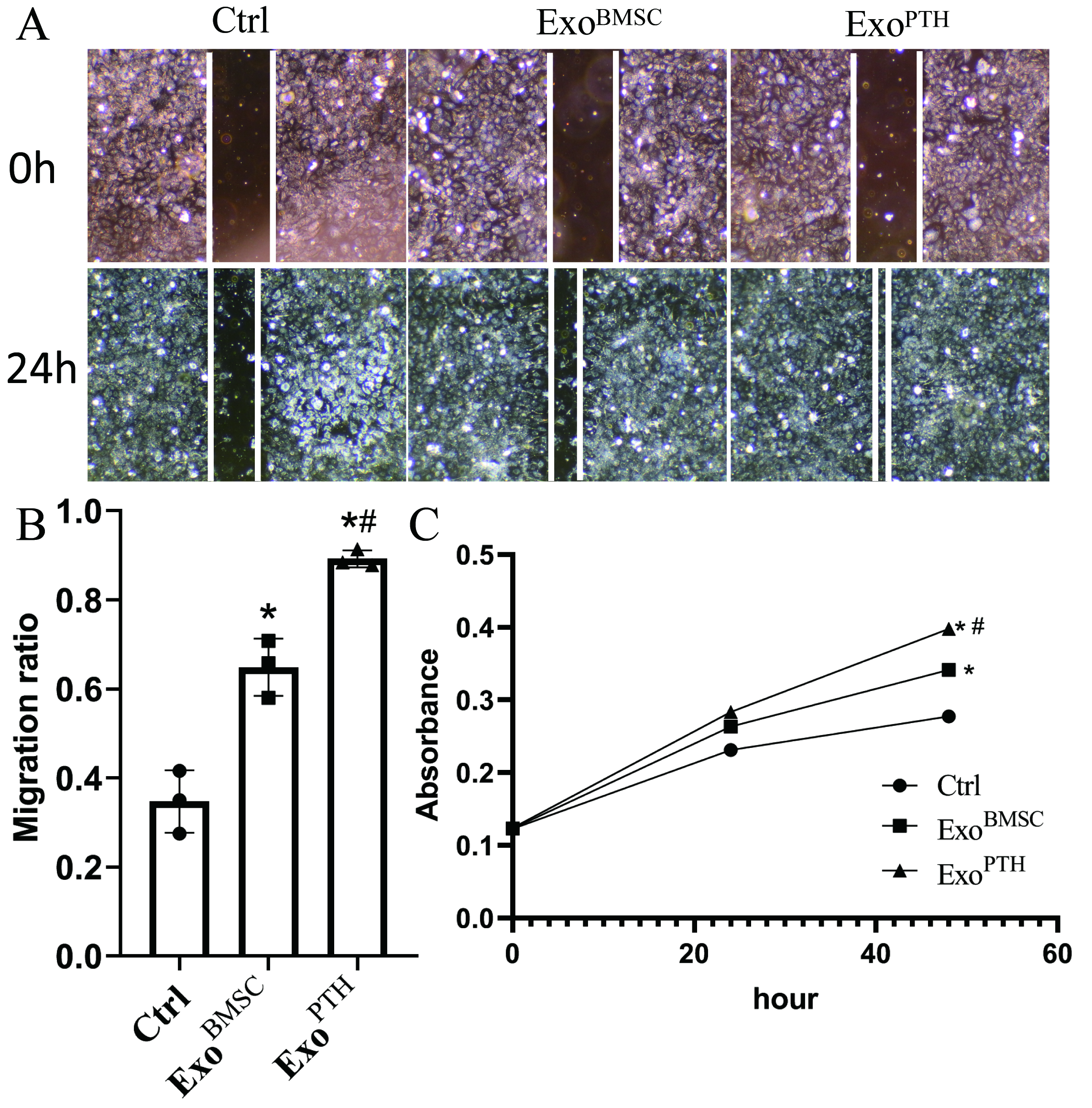

Supplement: Supplementary file 3 — Additional file 3: Figure Supplementary 3. The effect of ExoBMSC and ExoPTH on chondrocyte proliferation was assayed by the scratch wound-healing migration assay and CCK-8. (A). The scratch wound-healing migration assay was performed with control, ExoBMSC, and ExoPTH groups. (B). Statistical results of the migration ratio. (C). The cell proliferation ability of the control, ExoBMSC and ExoPTH groups was detected by a CCK-8 assay. Data are presented as the mean ± SD, *p < 0.05 versus the control group and #p < 0.05 versus the ExoBMSC group. ExoBMSC: exosomes derived from BMSCs; ExoPTH: exosomes derived from PTH-preconditioned BMSCs. [file 13075_2022_2778_MOESM3_ESM.tif]

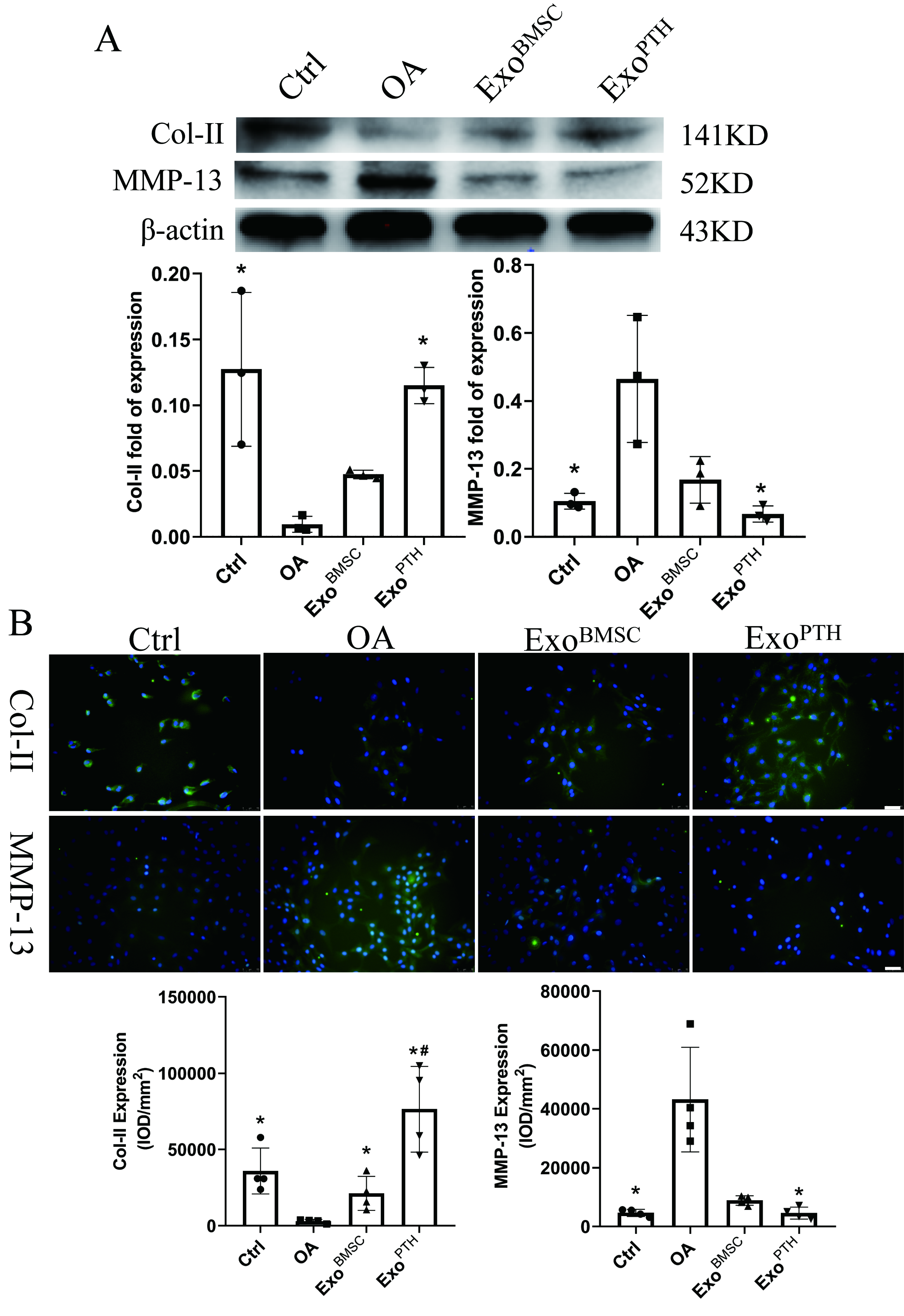

Supplement: Supplementary file 4 — Additional file 4: Figure Supplementary 4: The effect of ExoBMSC and ExoPTH on the OA chondrocyte extracellular matrix was detected by Western blot and immunofluorescence. (A). Western blot assay for collagen-II (Col-II) and matrix metalloproteinase-13 (MMP-13) in each group. (B). Immunofluorescence assay for Col-II and MMP-13 in each group. Data are presented as the mean ± SD, *p < 0.05 versus the OA group and #p < 0.05 versus the ExoBMSC group. Bars = 50 μm; OA: osteoarthritis; ExoBMSC: exosomes derived from BMSCs; ExoPTH: exosomes derived from PTH-preconditioned BMSCs; BMSCs: bone marrow mesenchymal stem cells. [file 13075_2022_2778_MOESM4_ESM.tif]
