# Supplementary material for: PTH (1-34) enhances the therapeutic effect of bone marrow mesenchymal stem cell-derived exosomes by inhibiting proinflammatory cytokines expression on OA chondrocyte repair in vitro
Source: Arthritis Res Ther. 2022 Apr 29;24:96. doi: 10.1186/s13075-022-02778-x (PMC9052609; doi:10.1186/s13075-022-02778-x)
Supplement: Supplementary file 5 — Additional file 5: Table 1. List of the three PCR Primer Pairs chosen for in vitro PCR validation. [file 13075_2022_2778_MOESM5_ESM.zip › 1-Supplementary table 1.doc]

### Table 1.

### List of the three PCR Primer Pairs chosen for in vitro PCR validation

| Name | Forward primer | Reverse primer |
| --- | --- | --- |
| β-actin | 5′-CTGTGTTGTCCCTGTATGCCTCTG-3′ | 5′-GGAACCGCTCATTGCCGATAGTG-3′ |
| IL-2 | 5′-TGAGCAGGATGGAGAATTACAGG-3′ | 5′-GTCCAAGTTCATCTTCTAGGCAC-3′ |
| IL-6 | 5′-CCAAGAGGTGAGTGCTTCCC-3′ | 5′-CTGTTGTTCAGACTCTCTCCCT-3′ |
| TNF-α | 5′-GACGTGGAACTGGCAGAAGAG-3′ | 5′-TTGGTGGTTTGTGAGTGTGAG-3′ |
| ROS1 | 5′-ATCCGAGTGGTGGTGGTCTATACG-3′ | 5′-ATTTGACGCTACTGCTCTGAACTGG-3′ |
